# Understanding the individual to implement the ecosystem approach to fisheries management

**DOI:** 10.1093/conphys/cow005

**Published:** 2016-04-07

**Authors:** Taylor D. Ward, Dirk A. Algera, Austin J. Gallagher, Emily Hawkins, Andrij Horodysky, Christian Jørgensen, Shaun S. Killen, David J. McKenzie, Julian D. Metcalfe, Myron A. Peck, Maria Vu, Steven J. Cooke

**Affiliations:** 1 Fish Ecology and Conservation Physiology Laboratory, Department of Biology, Carleton University, 1125 Colonel By Drive, Ottawa, ON, CanadaK1S 5B6; 2 Department of Biology, University of Ottawa, 30 Marie-Curie Private, Ottawa, ON, CanadaK1N 9B4; 3 Department of Marine and Environmental Science, Hampton University, Hampton, VA 23668, USA; 4 Department of Biology and Hjort Centre for Marine Ecosystem Dynamics, University of Bergen, PO Box 7803, Bergen 5020, Norway; 5 Institute of Biodiversity, Animal Health, and Comparative Medicine, Graham Kerr Building, University of Glasgow, Glasgow G12 8QQ, UK; 6 Equipe Diversité et Ecologie des Poissons, UMR5119 Ecologie des Systèmes Marins Côtiers, Université Montpellier, Place Eugène Bataillon, Montpellier cedex 5 34095, France; 7 Centre for Environment, Fisheries and Aquaculture Science (Cefas), Lowestoft Laboratory, Suffolk NR33 0HT, UK; 8 Institute of Hydrobiology and Fisheries Science, Center for Earth System Research and Sustainability, Olbersweg 24, Hamburg 22767, Germany

**Keywords:** Conservation behaviour, ecosystem-based management, ecosystem models, fisheries management, individual-based models, individual-level variation

## Abstract

Ecosystem-based approaches to fisheries management (EAFMs) have emerged as requisite for sustainable use of fisheries resources. At the same time, however, there is a growing recognition of the degree of variation among individuals within a population, as well as the ecological consequences of this variation. Managing resources at an ecosystem level calls on practitioners to consider evolutionary processes, and ample evidence from the realm of fisheries science indicates that anthropogenic disturbance can drive changes in predominant character traits (e.g. size at maturity). Eco-evolutionary theory suggests that human-induced trait change and the modification of selective regimens might contribute to ecosystem dynamics at a similar magnitude to species extirpation, extinction and ecological dysfunction. Given the dynamic interaction between fisheries and target species via harvest and subsequent ecosystem consequences, we argue that individual diversity in genetic, physiological and behavioural traits are important considerations under EAFMs. Here, we examine the role of individual variation in a number of contexts relevant to fisheries management, including the potential ecological effects of rapid trait change. Using select examples, we highlight the extent of phenotypic diversity of individuals, as well as the ecological constraints on such diversity. We conclude that individual phenotypic diversity is a complex phenomenon that needs to be considered in EAFMs, with the ultimate realization that maintaining or increasing individual trait diversity may afford not only species, but also entire ecosystems, with enhanced resilience to environmental perturbations. Put simply, individuals are the foundation from which population- and ecosystem-level traits emerge and are therefore of central importance for the ecosystem-based approaches to fisheries management.

## Abstract (precis)

Variation of physiological and behavioural traits among individuals within a population is an important factor for ecosystem function, and correspondingly, ecosystem approaches to fisheries management (EAFM). Here, we review instances of individual level variation in fishes with practical guidance for EAFM.

## Introduction

Nearly every biology textbook begins with a reductionist overview of biological hierarchies, emphasizing the long-standing and axiomatic nature of this concept (Simon, 1962). Molecules, genes, cells, tissues and organs collectively form individuals, an association often referred to as biological organization (Dobzhansky, 1964). In turn, individuals form the basis of populations, communities and ecosystems, referred to as ecological organization (Peterson, 2000). Scientists often concentrate on one or several levels within the complex biological hierarchy in an attempt to provide focus and yield tractable questions (Valentine and May, 1996). This does not mean that what happens at one level is not important to another, as emergent properties at one level may be manifested collectively at a higher level (Korn, 2005). Theoretically, one could link ecosystem changes back to processes that occur at the level of the molecule or gene or vice versa, although such scaling is not straightforward (Cooke et al., 2014a) and requires complex models and assumptions about mechanistic processes and links across hierarchical levels (Nisbet *et al.*, 2000; Benfey and Mitchell-Olds, 2008; Forbes *et al.*, 2008).

For those who work within the realm of natural resource management and conservation, it is apparent that environmental or ecological problems often cascade across multiple levels of biological and ecological organization. For example, various environmental factors (see the Fry paradigm; Fry, 1947) influence cellular processes and organ function and collectively influence individual fitness (Calow, 1989). Individual responses to environmental variation are limited by physical, physiological and phylogenetic mechanisms (Fry, 1947; Ricklefs and Wikelski, 2002). Individual fitness then drives population-level processes (Calow and Forbes, 1998; Maltby, 1999; Ricklefs and Wikelski, 2002), which can influence ecosystem structure and function (Helmuth, 2009). In this context, individual physiological abilities and tolerances are the transfer functions that directly link organisms and, eventually, populations to their environment (Fry, 1971; Weissburg and Browman, 2005; Jusup *et al.*, 2011; Horodysky *et al.*, 2015).

In many natural systems, the effects of anthropogenic and natural stressors on ecosystem interactions are becoming increasingly apparent (e.g. Crain *et al.*, 2008). Consequently, resource management strategies are moving towards holistic approaches encompassing a broad array of ecosystem variables (Brunner and Clark, 1997). This is particularly salient in the fisheries management realm, where regulatory bodies have embraced the concept of an ecosystem approach to fisheries management (EAFM), albeit with varying degrees of commitment and application (Arkema *et al.*, 2006; Pitcher *et al.*, 2009; Patrick and Link, 2015; Skern-Mauritzen *et al.*, 2015). Although discrepancies in terminology exist and objectives vary widely (Yaffee 1999; Garcia *et al.*, 2003; Arkema *et al*., 2006), in this article we adopt a general characterization of EAFM *sensu* Hilborn (2011) as a set of contemporary management strategies made at the level of the fish stock (e.g. bycatch mitigation and habitat modification) which incorporate emerging tools providing an ecosystem context to those actions (e.g. predator–prey interactions and physical oceanographic modelling). We limit our scope to conventional management jurisdictions and do not presume to address all anthropogenic impacts within the broader realm of ecosystem-based management (e.g. terrestrial land-use policies); however, the consequences of such extrinsic factors for fisheries management bear similar deliberation. The premise of our arguments centres on the tenet that EAFM aims to maintain the natural ecological function and evolutionary stability of a particular population, to accommodate sustainable fisheries (Pikitch *et al.*, 2004).

At the same time that the EAFM gains traction with natural resource agencies and resource managers (Brunner and Clark, 1997; Garcia and Cochrane, 2005; Patrick and Link, 2015), there is a growing recognition that individual trait diversity is a pervasive form of population-level variation with ecological implications (Spicer and Gaston 1999; Bolnick *et al*., 2003). In particular, inter-individual diversity in physiology and behaviour may affect the persistence and resilience of a population to disturbances such as fishing pressure, habitat loss and alteration, changes in prey base, and climate-driven warming and ocean acidification. Phenotypic diversity is also relevant to ecosystem interactions (e.g. predator–prey relationships, trophic cascades, sexual selection and habitat selection; Miner *et al.*, 2005; Wellenreuther *et al.*, 2014) and plays a mediating role in the genetic consequences of managing populations (e.g. gear selectivity, and fisheries-induced evolution; Kuparinen and Merilä, 2007). As holistic management frameworks continue to be adopted, it is important for managers and researchers to consider the effects of human activity on fishes at an individual level and attempt to reconcile the challenges of scaling information and concepts from individuals to ecosystem (Helmuth 2009; Cooke *et al.*, 2014a). Emerging tools of conservation physiology and conservation behaviour enable the monitoring and evaluation of interpopulation variance and may provide a basis for informing future management practices (Cooke *et al.*, 2014b).

**Figure 1:**
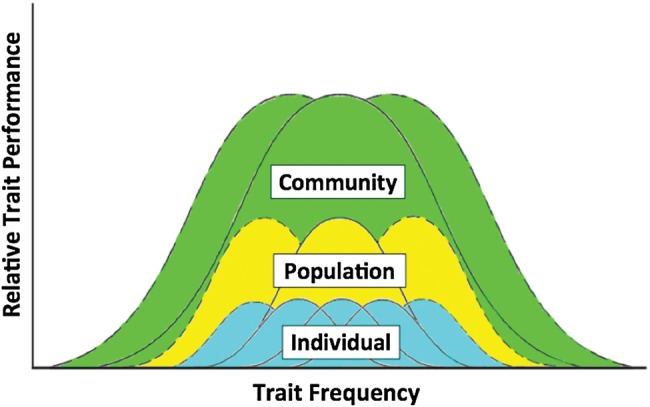
Conceptual depiction of trait diversity within a community, noting that the mean value (performance or optima) of a trait (continuous lines) measured at the population level can be composed of an array of constituent phenotypes at the individual level, the performance of which can vary across individuals through space and time (i.e. the portfolio effect). Both natural and artificial selection (whether direct or indirect) influence the performance of individual-level traits, which may result in cascading effects upwards through the ecological hierarchy (dashed lines).

Holistic approaches to fisheries management are increasingly becoming the norm (Curtin and Prellezo, 2010), although in practice a number of challenges remain (Meyer and Swank, 1996; Brunner and Clark, 1997; Schindler and Hilborn, 2015; but see Patrick and Link, 2015). It has long been acknowledged that extensive variation exists among populations [see Prosser (1955) and the Beverton-era work on life-history variation across fish populations]. Such variability has been embraced by fisheries management agencies as embodied by the ‘stock’ concept (Berst and Simon, 1981) and evolutionarily significant management units (Allendorf, 1995). For many of the most commercially valuable species or those of conservation concern, such as Pacific salmon (*Oncorhynchus* spp.), Atlantic cod (*Gadus morhua*) or bluefin tuna (*Thunnus thynnus*), fisheries management has defined biologically discrete stocks, which are separate entities when it comes to monitoring, setting quotas, harvesting and reporting catches. While accounting for demographic shifts at the population and sub-population levels (e.g. size–frequency distribution) is the classic method of fisheries monitoring, ecologically significant sources of variation occur within a population (e.g. standing rates of trait variation). The phenotypic variance within a population can be achieved through different modes (i.e. the portfolio effect; Bolnick *et al.*, 2011), with the potential for artificial (harvest) selection to act upon discrete traits, thus driving local shifts in phenotype abundance or performance (Fig. 1; Schindler *et al.*, 2010).

Failing to account for inter-individual variation within stocks can place management objectives at risk. The individual is an observational unit of value for resource managers and may be particularly informative when the objective is to understand ecosystem-scale processes (Nisbet *et al*., 2000; Cooke *et al.*, 2014b). Here, we contend that not only population-level but also individual variation is one of the benchmarks against which EAFM actions should be based. We call on resource managers to consider incorporating the growing body of knowledge pertaining to individual diversity within populations, which can enhance EAFM by providing greater insights into the cryptic and sub-lethal effects of human activity and environmental change. We provide examples of how individual trait diversity is a crucial component of ecosystem processes, structure and function and, hence, EAFM. We conclude with recommendations on the use of individual-level information in ecosystem approaches and identify areas of need moving forward.

Although sexual differences within a species often relate to ecologically relevant parameters, such as life history, behaviour and physiology, we limit our exploration of sex-based differences because these have already received attention elsewhere (Rowe and Hutchings, 2003; Hanson *et al*., 2008).

## Individual trait diversity in fishes

### Life history

**Figure 2:**
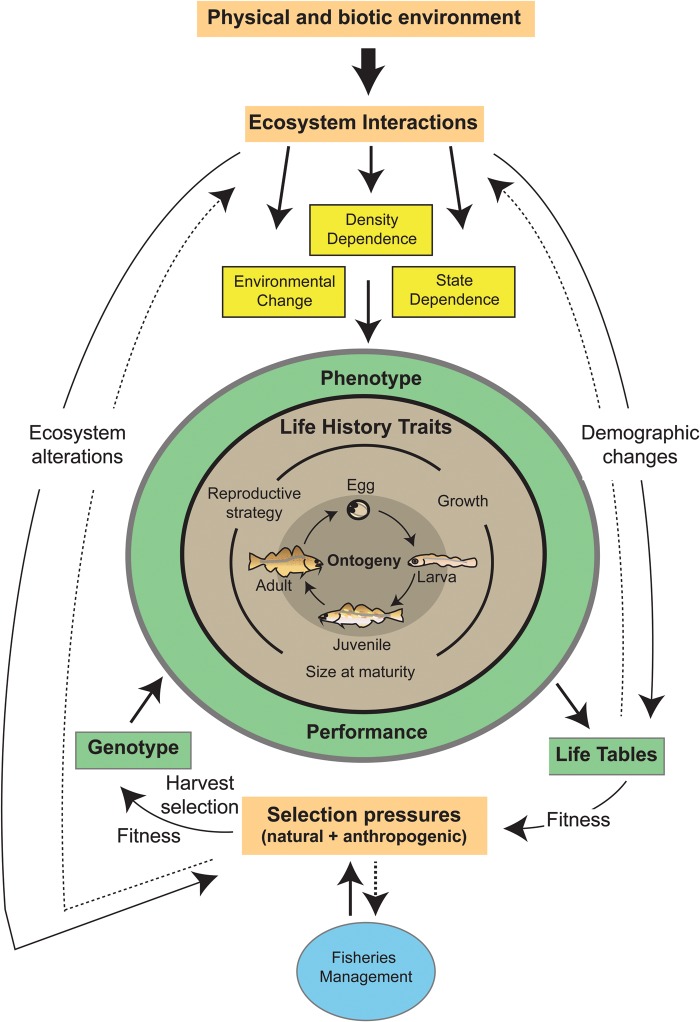
Conceptual depiction of the interaction between fisheries management (blue circle) and ecosystem processes (adapted from Ricklefs and Wikelski, 2002). Individual attributes and variation therein (grey circle) give rise to a phenotype for which performance (green circle) is influenced by ecosystem interactions (orange boxes). Feedbacks between environmental factors (yellow boxes) are mediated by ecosystem alterations and demographic change (continuous and dotted lines).

There is a longstanding recognition within fisheries science, pioneered by the work of Ray Beverton, that the array of life-history traits across many commercial fish stocks can be dynamic (Beverton and Holt, 1959; reviewed by Shuter and Abrams, 2005). The causes and consequences of such variation have received considerable attention in the intervening decades, with the consensus that anthropogenic environmental change, including harvest pressure, can induce shifts in life-history traits within fish populations (Conover *et al.*, 2005; Kuparinen and Merilä, 2007; Palkovacs *et al.*, 2012). Diversity in life-history traits within a population is an important consideration for EAFM, because it connects multiple facets of inter-individual diversity (Fig. 2), including ontogenetic processes, such as growth rate, size at maturity and natural mortality, in addition to phenological patterns related to sex determination, migration timing and reproductive strategies (Pukk *et al.*, 2013). The dynamics of a population are influenced by the diversity its phenotypes, as shaped by genetic and environmental factors, and maintenance of this variation provides resilience and adaptability to changing environments (Ricklefs and Wikelski, 2002; Winemiller, 2005). It is worth noting that life-history traits can be viewed as emergent properties of the evolutionary and environmental forces governing individual development and performance (Roff, 2002) and that these same processes are implicated in virtually all examples of trait variation. As suites of traits are often correlated (Young *et al*., 2006; Conrad *et al*., 2011), a particular life-history trait often has associated physiological and behavioural characteristics, a concept that is revisited in the following sections and is of crucial importance for management decisions (Box 1).

Box 1: **Management implications of individual trait diversity**

Human activity has the capacity to influence the phenotypic diversity of wild animal populations (Wennersten and Forsman, 2012), with knock-on consequences for ecosystem function (Palkovacs *et al.*, 2012). Here, we present examples of how anthropogenic activity related to fisheries can both directly and indirectly influence individual trait diversity, the associated impact of this change on ecosystem processes, and the implications for management decisions. The examples selected represent a breadth of management scales, including practices that are directly related to an ecosystem approach to fisheries management (EAFM) policy, initiatives approached from an ecosystem-based management perspective, and broad-scale human-induced environmental change. From these examples, a common theme appears to emerge, whereby anthropogenic shifts in trait diversity in a population need to be monitored under an EAFM.

**Size-selective harvest**

In many fisheries, large (typically older) and subsequently higher-value individuals are preferentially removed from a population, although quotas and various size-based limits are often in place to promote sustainable harvest (Hutchings, 2009). Beyond the well-known population-level changes associated with removal of large, mature individuals, such as reductions in mean size at maturity and age at maturity, concomitant reductions in fecundity, and elevated natural mortality levels (Swain, 2011; Enberg *et al.*, 2012), the effects of these trait shifts on ecosystem function are significantly less understood. Alteration of ontogenic regimens, or the ‘pace of life’ of a population, has been related to stock vulnerability to harvest (Juan-Jordá *et al.*, 2015) and is likely to influence community tropho-dynamics (Réale *et al.*, 2010). Furthermore, behavioural changes associated with life-history shifts are possible, such as changes to feeding strategy (Audzijonyte *et al.*, 2013), social structure, habitat use or the timing and duration of migrations. Recent evidence of stock rebuilding in north Atlantic cod (Rose and Rowe, 2015) highlights the importance of state-dependent behavioural expression, particularly as it relates to our ability to monitor wild populations. In this case, renewed detections of cod spawning aggregations in the Bonavista Corridor followed an increase in the condition of reproductive age-class individuals at inshore feeding areas, suggesting that off-shore reproductive migrations may be condition- or density-dependent behaviours (Enberg *et al.*, 2009). Management practices that do not account for life-history changes will be likely to overestimate long-term yields; it is therefore prudent that population demographic monitoring (a central component of stock assessment under EAFM) contributes to an understanding of a population’s phenotypic ‘portfolio’ (Schindler *et al.*, 2010; Carlson *et al.*, 2011; Kershner *et al.*, 2011). Although the consequences of trait change will vary by species, monitoring how specific traits (especially those relating to body size) respond to selective regimens and environmental change will be instrumental in advancing our ability to manage at an ecosystem scale.

**Marine protected areas**

The designation of no-take marine protected areas (MPAs) may result in alterations to the phenotypic diversity and spatial structure of a population. A widely noted ‘spillover effect’ (Sale *et al.*, 2005), resulting in increased species diversity beyond the designated boundaries of the MPA, implies a strengthening of individual abundance and diversity; however, the effects on individual behaviour within and outside of MPAs are less understood. Two potential effects are the increased ‘catchability’ of gear-naïve fish near the boundaries of MPAs (Alós *et al.*, 2015) and the selection against long-distance dispersers within a population (Di Franco *et al.*, 2012). Although MPAs may protect a buffer population that can contribute to population recruitment outside its boundaries (i.e. the ‘rescue effect’), selective removal or differential performance of migrants may alter the phenotypic subsidy of the sink population (Burgess and Marshall, 2011). It is therefore pertinent that a population’s diversity in space use be accounted for in spatially explicit management strategies. Protecting those areas outside of MPAs that provide metapopulation connectivity and facilitate gene flow has been recognized as crucial to MPA efficacy and performance (Di Franco *et al.*, 2012; Wright *et al.*, 2015). This may be particularly salient for supporting the transfer and maintenance of transient phenotypes, such as exploratory individuals or super dispersers (Cote *et al.*, 2010; Marshall *et al.*, 2010), and is necessary to facilitate the above-mentioned ‘rescue effect’ conferred by MPAs. Further monitoring of trait diversity and the environmental constraints of migratory behaviours (i.e. density or state dependence) is recommended to understand the effect of MPA designation for stocks under EAFM.

**Climate change**

Human-induced climate change continues to alter physical environmental conditions on regional scales (IPCC, 2014), rapidly changing animal phenology and selective regimens (Carroll *et al.*, 2007) and presenting an important consideration for EAFM. Among the frequently noted impacts of a changing climate are rising sea surface temperatures and ocean acidification, which have received considerable attention for their ecological consequences (Munday *et al.*, 2009a). Rising temperatures have the capacity to alter the primary productivity of ecosystems, influencing bioenergetic relationships on individual and ecosystem scales (Barneche *et al.*, 2014; Queirós *et al.*, 2015). In terms of individual fish diversity, metabolic constraints on organismal performance may result in homogenized phenotypes via directional selection or portfolio dampening (Helmuth, 2009; Holt and Jørgensen, 2014). Changes to regional climate may also induce shifts in the phenology, an important constraint of ecosystem processes of relevance to EAFM. Earlier spawning migration during periods of warming is a widely documented population-level phenomenon among salmonids; however, recent investigations have contrasted the relative rate of phenotypic change both across and within species and life-history strategies to reveal the ecological processes underlying phenological change (Kovach *et al.*, 2013). The authors highlighted that the response to climate warming (here, migration timing) was consistently earlier across species; however, the largest shift in phenotypic variation was seen within populations (regardless of an individual’s life-history strategy). However, the disparate responses observed by Kovach *et al.* (2013) between some life-history strategies of the same population denote an aspect of population ecology, known as ‘biocomplexity’, by which discrete phenotypes can respond to differing environmental conditions. Given the dynamic shifts in phenotypic expression resulting from climate change, an important management objective remains to parse the genetic mechanisms underlying this change. Plastic responses are likely to be widespread (Enberg *et al.*, 2009) but not ubiquitous. Further investigation is required to determine the heritability and ecological processes responsible for changing traits at a population level, particularly in the face of continued environmental change.

Typically, life-history traits are regarded as a property of populations, with acknowledgement of some variance among individuals through space and time. There is, however, increasing recognition of the degree of standing diversity within a population (Ricklefs and Wikelski, 2002; Bolnick *et al.*, 2011; Modlmeier *et al.*, 2014). For example, variation in the timing of important ontogenic events, such as maturation and reproduction, has been noted within and across populations (Réale *et al.*, 2010), with consequences for vulnerability to decline from harvest (Juan-Jordá *et al.*, 2015). Aside from direct harvest, indirect anthropogenic habitat alteration has also been found to influence life-history diversity in fish populations (Giery and Layman, 2015; Riesch *et al.*, 2015). Thus, while life-history dynamics can be observed at the population level, shifts in phenotypic diversity are manifested at the individual level and can occur in short time scales.

### Body size

Morphological characteristics provide an accessible basis for evaluating among-individual variation and have a long history of use in fisheries management. The body size of a fish is a function of sex, life-history traits, ontogeny, energetics and genetics (Stearns, 1983; Winemiller and Rose, 1992) and relates to a variety of physiological and behavioural traits, in interaction with the abiotic and biotic environment. Mean body size, as well as mean size at maturation, are central parameters in fisheries management frameworks, often employed for stock assessments in data-deficient applications (Andersen and Beyer, 2013). In many instances, however, variation of these traits exists within a population, providing an ecologically relevant source of data for individual variation within a population.

Perhaps the greatest influence of the body size of an individual on physiological variables relates to energetics. Given that an organism must acquire resources and partition available energy among demands for maintenance, activity, growth and reproductive maturation, body size and metabolic processes can impose a constraint on fitness (Barton, 2002). Metabolic rates scale with mass and temperature (Heusner, 1982), providing two sources of size-based individual variation. Larger fishes benefit from negative allometric relationships, because they expend proportionally less energy for vital functions compared with smaller fishes (Heusner, 1982). Likewise, differences in swimming efficiency correspond to body size, where larger fishes incur lower mass-specific energetic costs (Schmidt-Nielsen, 1972). However, a number of studies have shown intraspecific deviation from the universal scaling exponent (*b* = 0.75; Heusner, 1982), with several examples found in fishes (Bokma 2004; Glazier, 2005; Killen *et al.* 2007, 2010; Burton *et al.* 2011). Importantly, larger individuals typically have energetic and ecological advantages that increase fecundity and, in some cases, investment of reproductive energy (Birkeland and Dayton, 2005). In some species, offspring of larger individuals also exhibit improved fitness characteristics, with examples including more rapid maturation, decreased sensitivity to food deprivation (Berkeley *et al.*, 2004), and avoiding size-dependent predation. Recent stock recruitment models have found, using asymptotic growth rate and assuming increased offspring success for larger individuals, that fisheries reference points are not likely to be impacted significantly for most stocks (Andersen and Beyer, 2013). Notably, however, these characteristics are likely to be more significant for longer-lived, slower-maturing species, many of which constitute vulnerable fisheries, highlighting the differential response of traits across species.

The size of an individual also gives rise to many individual-level behavioural differences in fishes. In social species, variation in body size can influence hierarchical structures; larger fish are typically dominant in competition for resources and mates (Bisazza *et al.*, 1996; Yue *et al.*, 2006; Kohda *et al.*, 2008), often conferring increased foraging and reproduction but subject to trade-offs with survival (Enberg *et al.*, 2012). Schooling behaviours in fish can be determined in part by body size (Jacob *et al.*, 2007); it has been documented that herring (*Clupea harengus*) and mackerel (*Scomber scombrus*) sort according to size, with individuals choosing neighbours of similar size (Pitcher *et al.*, 1985). Variation in body size also influences predator–prey dynamics; it is energetically advantageous to have a larger body size as a predator, in order to consume smaller prey more efficiently, whereas prey with a larger body size can avoid gape-limited predators (Cohen *et al.*, 1993). A study on three-spined stickleback (*Gasterosteus aculeatus*) found, however, that when not gape limited, predators tended to select larger prey items (Gill and Hart, 1996).

### Physiological capacity, tolerances and performance

It has been suggested that physiology acts as a filter or transfer function between the environment and fitness (Ricklefs and Wikelski, 2002; Horodysky *et al.*, 2015), wherein an individual’s tolerance and response to external stressors are often indicative of a population’s long-term persistence and distribution (Fry, 1947; Chown, 2012; Seebacher and Franklin, 2012; Cooke *et al.*, 2013). Although fitness of the individual is the ultimate consequence, environmental impacts may be mediated at lower cellular levels and can even be tissue specific (Johnson and Tricker, 2010). By examining energy flow between organisms and their environment, in addition to mechanistic alterations at genetic and biochemical levels, physiological systems provide insight into variation among individuals’ responses to their environments (Carey, 2005). One striking example of this is the diversity of endocrine activity among individuals, and how individual responses to environmental challenges vary, including variability in hormone production, receptor activity and signalling pathways (Williams, 2008), as well as changes to gene expression (Richards *et al*., 2010). In response to environmental stressors, the regulation of these endocrine systems may stimulate stress responses or inhibit reproduction by targeting the stress axis or hypothalamic–pituitary–gonadal axis, respectively (Barton, 2002; Romero, 2004). Physiology can, therefore, be used as a tool to assess the degree of stress exerted on individuals by their environments and the resulting consequences for reproductive potential (Wikelski and Cooke, 2006).

Investigations of physiological performance have highlighted the degree of inter-individual variability in fishes. In particular, bioenergetic analyses of aerobic scope for activity, swimming performance and associated metabolic processes have provided insights into the causes and consequences of variation within populations (Kolok and Farrell, 1994; Oufiero and Garland, 2009; Marras *et al.*, 2010). Variations in metabolic scope and routine metabolic rates may also influence ecologically significant behaviours (Burton *et al.,* 2011), such as risk taking to secure resources (Killen *et al*., 2011), positioning within schools (Killen *et al.* 2012a) and dominance (Reid *et al*., 2012; Killen, 2014). Likewise, metabolic demand (for compensatory growth) has been found to influence routine metabolic rate, thermal preference and activity, illustrating the relationship between physiology and behaviour (Killen, 2014).

Variation in individual tolerance to environmental parameters, such as temperature, hypoxia, ammonia, salinity, pH and CO_2_, has also been documented (Munday *et al.*, 2009a,b; Killen *et al.* 2012b) and, through differential mortality, may also present a selective filter, impacting ecological and evolutionary processes (Munday *et al.*, 2012). Genetic heritability of physiological traits, and thus evolutionary potential in the face of changing environments, has also been documented. For example, thermal tolerance of offspring from the same female but different males (sires) can differ (Politis *et al*., 2014). In a second example, cardiac performance and thermal tolerance are positively related within individuals but highly variable between families of Atlantic salmon (*S. salar*; Anttila *et al.*, 2013). There is also evidence to suggest that sexual dimorphism in physiological traits exists, including variable stress responses between sexes of many sharks (*Elasmobranch* spp.), males having consistently higher levels of circulating corticosteroids than females (Anderson, 2012), and similar results have been reported in rainbow trout (*Oncorhynchus mykiss*; Pottinger *et al.* 1995; Pottinger and Carrick 1999). Rainbow trout also exhibit differences in cardiac metabolism between sexes, with females having a higher tolerance to hypoxia, whereas males have a greater capacity for aerobic and lipid metabolism (Battiprolu *et al.*, 2007). Among-individual variation in energetic performance and environmental tolerances may form the basis for spatiotemporal variability in habitat selection via behavioural thermoregulation (Huey, 1991; Horodysky *et al.*, 2015).

The relationship between environmental conditions, individual size and metabolism has given rise to the metabolic theory of ecology (Brown *et al.*, 2004) and the dynamic energy budget theory (Nisbet *et al.*, 2000). Dynamic energy budgets attempt to reconcile changes in fish growth (and reproductive allocation) with environmental changes through time and space. Generally, these models consider both abiotic factors (e.g. temperature) and biotic factors (e.g. food availability) and are thus regionally specific and species specific, with notable differences among and within species (Freitas *et al.*, 2010). Both models are now being used to explore ecosystem-scale relationships, using community data (Barneche *et al.*, 2014) and in conjunction with individual-based models (Beaudouin *et al.*, 2015).

### Behaviour

There exists a wide range of behavioural diversity in fishes that should be given consideration for fisheries management. When these differences in individual behaviour are consistent and repeatable, they are variously referred to as ‘animal personality’, ‘behavioural types’ or ‘behavioural syndromes’ and have been observed in many taxa, including fishes (Sih *et al.*, 2004; Dingemanse *et al.*, 2010; Conrad *et al.*, 2011). The behavioural variation can be a function of life-history stage and may often correlate with a suite of physiological variables which, together, contribute to an individual’s relative fitness (Buchholz, 2007; Cooke *et al.*, 2013) or life-history strategy (Réale *et al*., 2010). Behavioural studies on fishes have been prominent in our understanding of the bold–shy continuum (Sih *et al.*, 2004; Conrad *et al.*, 2011). For example, migrant and resident individuals can exist in sympatric populations leading to partial migrations and, in some instances, the probability of migration has been directly linked to the boldness of individuals, as seen in freshwater roach (*Rutilus rutilus*; Chapman *et al.*, 2011a). In addition, bolder individuals are typically more likely to engage in other risk-taking behaviours (Sih *et al*., 2004; Chapman *et al.*, 2011b). For example, elevated activity may improve foraging opportunity, but it may also increase predation risk (Lima and Dill, 1990; Cote *et al.*, 2010). This phenomenon has been linked to harvest vulnerability; Biro and Post (2008) reported that, independent of body size, bold, fast-growing rainbow trout were more vulnerable to a simulated commercial fishery than shy, slow-growing individuals.

Consistent behavioural variations may be linked proximally by physiological requirements and sensitivities and, ultimately, by environmental factors, such as predator or prey densities, and stress responses (Dall *et al.*, 2012; Killen *et al.*, 2013). In addition, available evidence also suggests that individuals within a species can show intrinsic behavioural diversity that can help to generate variations in body size and physiology in complex cause-and-effect feedbacks (Biro and Stamps, 2008, and references therein). This view is also supported by a theoretical model for the evolution of information use during decision-making in fish, where consistent behavioural types emerged as a result of ontogeny, sex and frequency-dependent pay-offs, and with consequences for growth, size and survival (Giske *et al.*, 2014).

There is also evidence to suggest that large pelagic sharks are prone to state-dependent constraints on migratory behaviour and movements related to reproduction. Evaluating large tiger shark energetics at a seasonal aggregation site, Gallagher *et al*. (2014) found a positive relationship between body condition and triglycerides, a source of energy to fuel challenging life-history phases. The authors found a high degree of individual variation in both measures and hypothesized that the low number of individuals exhibiting strong condition and high energy stores in space and time were disproportionately important to stock health. Individual differences also influence vulnerability to exploitation, as seen in trophy sport fisheries, where the largest and most fecund individuals are targeted. Using the shark example above, the same individuals that hold increased conservation value also experience disproportionate harvest pressure, leading to problems when the species in question is threatened (Shiffman *et al*. 2014).

## Synthesis: integrating individuals into ecosystem-based approaches to fisheries management

### Role of individuals in eco-evolutionary processes

Through experiencing and responding to their environment, individuals are the substrate upon which natural selection operates, acting as the interface between the population or species’ gene pool and the environment. Variation among individuals’ responses to environmental parameters is therefore a critical link that drives ecosystem dynamics (Fig. 2; Spicer and Gaston, 1999: Ricklefs and Wikelski, 2002). The ecological literature is replete with examples of individual diversity (reviewed by Bolnick *et al.*, 2003), and study of the ecological relevance of this diversity (Van Valen, 1965) arose in parallel with life-history examinations in the context of fisheries (Beverton and Holt, 1959). A renewal of interest in the ecological consequences of individual-level variation (including behavioural and physiological aspects) has been manifested by the formation of new theoretical frameworks within evolutionary ecology (Schoener, 2011; Dall *et al.*, 2012). Recent empirical and theoretical work has aimed at revealing mechanisms by which individual diversity and rapid trait change are driving ecological interactions and ecosystem function (Bolnick *et al.*, 2007; Ellner *et al.*, 2011; Lundsgaard-Hansen *et al.*, 2014). Drawing heavily on lessons from the fisheries realm, investigations have documented that individual trait variation plays a significant role in trophic ecology, ecosystem processes and evolutionary feedbacks (Palkovacs *et al.*, 2012). Bolnick *et al*. (2011) provide a framework for evaluating the ecological role of individual variation and have identified theoretical mechanisms, broadly categorized as direct and indirect effects, which operate at the individual level to affect ecosystem-scale processes. A main conclusion from this course of study is that the effect of trait change within a population may disproportionately influence ecosystem function and may even impart a greater effect than species extirpation or extinction (Palkovacs *et al.*, 2012). Some have taken this sentiment further and identified instances where the effects of a particular individual on population or community dynamics are so profound that the individual can be said to play a ‘keystone’ role in ecological processes (Modlmeier *et al.*, 2014).

### Eco-evolutionary dynamics of exploited fisheries

There is a breadth of literature in fisheries science that addresses observed trait change corresponding to overharvest (Conover *et al.*, 2005; Kuparinen and Merilä, 2007; Sharpe and Hendry, 2009; and see Box 1 for select examples). Managing fisheries resources at an ecosystem scale calls practitioners to account for ecosystem processes at evolutionary time scales (Pikitch *et al*., 2004; Laugen *et al*., 2014). For example, size-selective mortality is a central component shaping life histories, influencing the recruitment capacity of a population and its ability to buffer additional pressures, such as changing environments and adult predation (Kuparinen *et al.*, 2012). Human-induced selection is an important factor influencing mortality and is a crucial consideration in forecasting fisheries productivity (Kuparinen and Merilä, 2007; Gallagher *et al.* 2015). For example, in a model where risk taking was allowed to evolve in response to harvesting, natural mortality was predicted to increase by about half the imposed fishing mortality (Jørgensen and Fiksen, 2010; Jørgensen and Holt, 2013). Furthermore, it is widely recognized that commercial fishing practices can preferentially take larger individuals, potentially selecting for fish that mature earlier and at smaller sizes (Ernande *et al*., 2004; Jørgensen *et al*., 2009). More recent evidence also suggests that active and passive gears may select for different behavioural phenotypes (Diaz Pauli *et al*., 2015), and differences in traits associated with metabolism and swimming performance may translate into variation in susceptibility to capture by trawl (Killen *et al.*, 2015).

Evidence and theory suggest that management actions (e.g. moratoriums and length restrictions) in response to observed decreases in size at maturation and elevated natural mortality have often failed to reverse life-history evolution induced by fisheries (Enberg *et al*., 2009; Swain, 2011; Kuparinen *et al*., 2014). Consequently, populations may be slow to rebound from fisheries-induced evolution and, taken together, these micro-evolutionary processes have the capacity to alter ecological interactions (Palkovacs *et al.*, 2012), become self re-enforcing (Kuparinen *et al.*, 2014) and contribute to ecosystem change (Daskalov *et al*., 2007). It is therefore crucial that an EAFM act in a proactive and co-adaptive management capacity, to account for standing levels of trait diversity within a population and to apply management strategies at a time scale relevant to observed changes in trait diversity.

An ongoing challenge under EAFM will be to parse the mechanisms driving the observed trait changes in many fisheries and evaluate the relative contributions of genotypic and phenotypic variation (Benton *et al*., 2006). In theory, fisheries-induced evolution can occur if individual fish within a population vary in their vulnerability to capture, via differences in size, physiology or behaviour, and if these differences are heritable (Uusi-Heikkilä *et al*., 2008; Diaz Pauli *et al.*, 2015). However, another important consequence of intensive fishing is the large-scale removal of biomass from aquatic ecosystems that can change adaptive landscapes through density-dependent effects on food availability, competition and recruitment, with selective effects on life-history traits and the specific phenotypes that are then favoured (Enberg *et al*., 2009; Heino *et al*., 2013). For example, in a dense population with intense competition for resources, more aggressive phenotypes may have an advantage, whereas in sparse populations with little competition for available resources, aggression may be under weaker selection. An EAFM needs to consider how harvest changes the complexion of ecosystems and generates density-dependent effects on evolutionary trajectories of the resident fishes. Fishing will inevitably lead to some change in population size, even in sustainable fisheries, so it is important to understand better how such changes might affect which traits are exposed to natural and anthropogenic selection.

Inherent in holistic management frameworks is the recognition that an increased understanding of fisheries characteristics can modify selective fishing practices to maximize the ecological and evolutionary stability of managed ecosystems (Pikitch *et al.*, 2004; Patrick and Link, 2015). As argued previously, individual diversity is ubiquitous among wild populations and may reflect an evolutionarily stable strategy that buffers against changing environmental conditions and sudden demographic shifts and may facilitate micro-evolutionary processes (Spicer and Gaston, 1999; Williams, 2008; Dall *et al.*, 2012). To embrace this paradigm, managers are called to act within two predominant schools of thought about regulating the specificity of capture methods under an EAFM; these are more or less selectivity in fish harvest methods. Zhou *et al*. (2010) have posited that a less selective ‘balanced exploitation’ could mitigate negative effects of selective harvest by spreading harvest pressure across genotypes and populations. As we have reasoned, the maintenance of genetic and phenotypic diversity fits within the framework of EAFM; however, a trade-off to decreased capture selectivity within species is that there may also be decreased capture selectivity among species (Garcia *et al*., 2012), potentially leading to unwanted bycatch. For example, alterations to fishing procedures or gear types to reduce size selectivity within a species may also retain species across a broad range of sizes. A challenge associated with decreasing selection on particular phenotypes is how to achieve a compromise between inter- and intraspecific gear selectivity.

### Deriving ecosystem reference points from individual diversity

Accounting for the great many sources of individual variation within fisheries selectivity is a daunting task, but ignoring the existence and role of such diversity has unintended consequences, such as those associated with fisheries-induced evolution. Given the current status of EAFM application (Fogarty and Rose, 2013; Patrick and Link, 2015), it is unrealistic that all sources of intrapopulation diversity be accounted for in forecasting models or for regulating harvest of specific phenotypes. Following recent calls for applying the current state of knowledge to ecosystem monitoring (Schindler and Hilborn, 2015), we suggest that monitoring suites of important morphological, physiological and/or behavioural characters, similar to detailed records of life-history traits, be implemented to derive benchmarks for individual diversity within populations. Detailed records of population trait change over time have been instrumental in casting light on contemporary processes (i.e. fisheries-induced evolution), and these data sets may also be leveraged to derive reference points for how shifts in the magnitude of within-population phenotypic diversity are important for ecosystem-scale processes. For example, Andersen and Beyer (2013) applied novel ecological concepts of maternal effects to re-evaluate reference points for northern cod, showing that life-history strategy was an important driver of harvest vulnerability.

Not only will existing reference points change as a function of fishing-induced evolution (Enberg *et al.*, 2010; Heino *et al.*, 2013), but there will also be a need for developing new reference points for fisheries management (e.g. Hutchings, 2009) to monitor, understand and conserve physiological, behavioural and phenotypic diversity better in fisheries populations. However, owing to the dynamic nature of ecosystems, characterizing a baseline ecosystem reference state may be difficult (Pauly 1995; Norris 2000), and the same may be true for individual variability. Consequently, reference points should be monitored consistently to account for and reflect shifting baselines.

Emerging technologies are enabling the monitoring of ecosystem and individual reference points with increasing precision and regularity. Alongside our growing understanding of abiotic and biotic environmental variables, it may be pertinent to develop individual-based metrics of ecosystem state. Although the development of ecosystem indicators based on individual organismal state presents a challenge, it is not an unattainable goal (Cooke and Suski, 2008; Adams and Ham, 2011; Jeffrey *et al.*, 2015). Understanding the spatial ecology of fisheries species and prey resources remains an integral challenge facing EAFM. In order to gain insights into individual diversity in habitat use over time, it is necessary to determine both the internal (behavioural and physiological) constraints and the external pressures (seasonality, climate variability and anthropogenic habitat change) governing space use (Nathan *et al.*, 2008). However, the free-ranging, predatory nature of many marine fish species introduces a number of difficulties for conducting individual-based studies. Observation-based studies for marine species can be expensive and highly demanding, although information produced from these studies can be directly relevant to fisheries management (e.g. Brill, 1994). In recent decades, technological advances, particularly in electronic data-storage tags, biologgers and biotelemetry technologies, have made long-term, high-resolution behavioural studies feasible (Hussey *et al.*, 2015). Furthermore, integrative studies that attempt to identify relationships between environmental parameters and internal physiological states will advance our knowledge of individual behaviour and its bearing on broader ecosystem processes. Behavioural traits associated with predation risk may be of particular importance for EAFM. This is because many of these traits, such as individual boldness, activity and exploratory tendency, could make some individuals especially likely to encounter and be captured by recreational or commercial fishing gears (Uusi-Heikkilä *et al*., 2008; Diaz Pauli *et al.*, 2015). As these traits also involve a trade-off with foraging activity, where riskier individuals are generally more active foragers (Lima and Dill, 1990), selection against a particular behavioural phenotype by fisheries could therefore affect species in other parts of the food web. Although large changes in fish biomass caused by fishing may outweigh shifts in phenotype abundance, in terms of the overall effects on the foraging requirements of a population and prey abundance, a depletion of top predators within an ecosystem will change selective pressures on resource prey and might influence the degree of risk-prone behavioural phenotypes present in prey populations (Abrams, 2009; Archard *et al*., 2012).

A significant challenge associated with EAFM, and specifically individual diversity-based reference points, is that not only do traits vary among individuals, but also the nature and strength of correlations between traits depends on the prevailing environmental conditions (Killen *et al*., 2013). For example, metabolic rate and growth rate may be positively correlated in the presence of high food availability but show weak or negative correlations when food is scarce (Burton *et al*., 2011). Genetic correlations among life-history traits are also labile across varying environments (Sgro` and Hoffmann, 2004). Efforts to preserve habitats or change suites of ecosystem components through habitat modification may therefore have unanticipated effects on which traits are exposed to direct or correlated selection. The ecosystem parameters that are defined as ‘normal’ or that are targets for preservation need careful consideration, because the environmental conditions will also have a direct bearing on the phenotypes that are expressed and the degree of variation that is present. In highly stable environments, for example, there may be less phenotypic heterogeneity on which selection can act, even if there is underlying genetic diversity within the population.

### Theoretical, strategic and tactical modelling in support of ecosystem-based approaches to fisheries management

Anthropogenic impacts on fisheries are not only manifested as predatory behaviour (Darimont *et al*., 2009, 2015), but mankind has also changed the atmosphere so that it in turn modifies the physical properties of the oceans (IPCC, 2014). In addition to harvest and climate change, humans also pollute, translocate species, spread pathogens, destroy or fragment habitats and reduce connectivity. It has therefore been argued that the selective environment has rarely, if ever, changed as rapidly as now (Carroll *et al.*, 2007). As such, there is a pressing need to parse the evolutionary response to changing selection pressures (e.g. Holt and Jørgensen, 2015), as well as the mechanisms of trait change within populations (Ellner *et al.*, 2011; Yamamichi *et al.*, 2011).

Models designed to study individual variation in adult fish often place less emphasis on a complex external environment and instead focus on life-history strategies or behaviours. The first models of population responses to changing selection pressures, such as industrial fishing, only had one or a few traits (e.g. Law and Grey, 1989), but models have since included evolutionary processes explicitly (Dunlop *et al*., 2009) or included more detailed physiology (Jørgensen and Fiksen, 2006). In these models, the level of between-individual variation emerged from physiological constraints, ecological processes and the selective environment. Two processes that may contribute to maintaining additive genetic variance are as follows: (i) fluctuating selection as a result of genotype–environment interactions (e.g. Grant *et al.*, 2004); and (ii) frequency dependence leading to coexistence of multiple strategies (Smith, 1982). Both are likely to be common and, when combined in a recent model for decision-making of behaviour in a pelagic fish, between-individual variation increased as one moved from fitness via life-history traits and behaviour through to the genome (Giske *et al.*, 2014). The authors’ interpretation is that there are several life-history combinations that successfully achieve fitness, several behaviours that over time can sustain similar life histories, and multiple ways for the genome to be coded that will result in the same behavioural decisions. The degree of between-level variation thus depends, in predictable ways, on the hierarchical level one focuses on. As a consequence, it is likely that one can make better or worse choices for quantifying between-level variance, particularly if the aim is to predict emergent population-level characteristics of interest to fisheries managers or conservationists.

It seems as though considerable changes are needed in classical fisheries models, which typically disregard within-population variation and instead lump individuals together in undifferentiated biomass (Beverton and Holt, 1959; Hilborn and Walters, 1992). Sometimes biomass is divided into age or length groups, and often sexually mature biomass is treated separately, but essentially the classical models treat individuals as being all the same and all doing the same things. Here, we highlight recent progress, in which individual variation plays a more central role. This is perhaps easiest to appreciate in individual-based models embedded within physical ocean models. Physical ocean modelling has, over the last decades, become a reliable and operationalized tool that assimilates observations and predicts temperature, flow and other water properties. For example, using a global ocean model, Follows *et al.* (2007) seeded 78 phytoplankton types that differed in their physiology and let the ambient environment and local competition for light and nutrients determine species abundance; the emerging biogeography recaptured many of the observed phenomena in species distributions and dynamics. Likewise, Mullon *et al.* (2002) simulated larval drift from multiple spawning sites and assumed natal homing by the adults and, after multiple generations of artificial evolution, the model had identified profitable spawning sites where the life cycle could be effectively closed. Another model study followed fish larvae of Atlantic cod that differed in their risk-taking behaviour (Vikebø *et al*., 2007); although intermediate risk taking was favoured, the optimal behaviour differed between spawning sites separated by only short geographical distances. In these studies, it is the dynamism of the local physical environment that brings about inter-individual differences in drift, growth, survival and success. By assuming physiology and behavioural rules, similar approaches are now used to predict inter-annual differences in the survival of early life stages for several commercial fish stocks (e.g. Daewel *et al*., 2011; Peck and Hufnagl, 2012). These individual-based models are designed to include individual trait diversity, but more work is needed to understand physical and biological processes (either bottom-up or top-down) acting to favour variation in specific traits of early life stages and how those and other traits may be linked and altered by the environment to influence individual (lifetime) fitness (Johnson and Hixon, 2010).

Currently, there are various types of complex models (beyond 0-D single- and multispecies models) that could incorporate information in inter-individual variation to inform EAFM. Most of these models represent the spatial dynamics of marine food webs, and some represent interactions among and between individuals and their environment. Biomass-based (Ecopath-with-Ecosim, EwE; Christensen and Walters, 2004), size-structured (Object-oriented Simulator of Marine ecOSystems Exploitation, OSMOSE; Shin and Cury, 2001) or ‘end-to-end’ (Atlantis; Fulton *et al*., 2011) simulations are examples of models that include spatially explicit food web interactions. Atlantis, the most complex model, creates a virtual ecosystem incorporating industrial activity, which allows trade-offs between various competing economic sectors to be examined, such as fisheries vs. conservation (Fulton *et al*., 2011; Morzaria-Luna *et al*., 2012). For each model, individual variability in key parameters could be included to account for ‘bottom-up’ ecosystem effects; for example, in EwE, model parameters such as diet matrices (prey availability) can be varied; in OSMOSE and Atlantis, movement variants of ‘super particles’ (individuals) can be included, or likewise for OSMOSE and Atlantis, aspects of life-history diversity such as changes in size at maturity or timing of ontogenic shifts can be included. Although these coding exercises may seem overly complex, recent studies have required similar considerations. For example, to capture long-term changes in the tropho-dynamic structure and function in Australian waters adequately, Atlantis simulations needed to include fisheries-induced evolution of maturation schedules and fish size (Fulton, 2011). Hence, food web models informing EAFM can be used to explore to individual-level variation and can demonstrate how failing to account for trait variation may affect management goals.

## Conclusion

Patrick and Link, (2015) state that, ‘ecosystem-based fisheries management takes a macrolevel look at system-level productivity, while protecting against overfishing, to smooth out the variability that occurs at the individual species level’. We contend that overlooking variation among individuals within a species or population can be a detriment to the objectives of EAFM. Implicit in their recognition of the drivers of population productivity (i.e. harvest selectivity, trophic interactions and fluctuating environmental conditions) is the role that individuals play in broader population responses to fisheries management. We agree that in the absence of perfect data or regulatory frameworks, managers should move forward within existing authorities to apply the current state of knowledge towards best management practices (Schindler and Hilborn, 2015) and argue that data for at least some examples of individual trait diversity are sufficient to do so (e.g. demographic size metrics). Promoting phenotypic diversity is an initiative that can impart fisheries with resiliency to environmental perturbations, as well as facilitating natural ecological and evolutionary processes. Furthermore, consideration of individual diversity not only falls within the scope of ecosystem approaches to fisheries management, but it is also consistent with the objective of stabilizing ecosystem processes.

It is not our intention to detract from the importance of multispecies perspectives and, indeed, we embrace the notion that holistic approaches need to consider ecosystem interactions from multiple viewpoints, including individual-level variation, in order to manage ecosystem processes effectively. As we broaden the scope of our management efforts to ecosystem scales, we call for the recognition that it is not only diversity in community structure, but also diversity within constituent populations that drives ecosystem function (Spicer and Gaston, 1999). In yet another instance of scaling across biological hierarchies, we can see that just as a broader tolerance of environmental constraints within an organism can increase its fitness, so can a diversity of individuals within a population raise the ecological plasticity of the community (Dall *et al.*, 2012; Lundsgaard-Hansen *et al.*, 2014). Given that supporting multiple ecological functions improves the resilience of an ecosystem to disturbance (Thrush and Dayton, 2010), it is imperative to provide a foundation for ecosystem variance rooted in diversity among individuals. There has been a recent call from academics that conservation practitioners should recognize the pitfalls of forecasting ecosystem states and, instead, implement management strategies that maximize ecosystem services across a range of future uncertainties (Patrick and Link, 2015; Schindler and Hilborn, 2015). We believe that current knowledge, regarding how individual diversity may underlie population responses to fisheries management, can support and enhance an EAFM by increasing ecosystem resilience in the face of environmental change.

## Funding

This paper is an output of the EU COST Action on the Conservation Physiology of Marine Fish. S.J.C. is supported by the Canada Research Chairs Program and the Natural Sciences and Engineering Research Council of Canada.
